# Breast cancers are rare diseases—and must be treated as such

**DOI:** 10.1038/s41523-017-0013-y

**Published:** 2017-04-11

**Authors:** John M. S. Bartlett, Wendy Parelukar

**Affiliations:** 1 Ontario Institute for Cancer Research, Toronto, Ontario Canada; 2 University of Toronto, Toronto, Canada; 3 University of Edinburgh, Edinburgh, UK; 4 Canadian Clinical Trials Group, Queen’s University, Kingston, Ontario Canada

## Breast cancers as rare diseases?

With over 1.5–1.7 million new diagnoses of breast cancer worldwide each year, and at a time when 25% of new cancers diagnosed in women annually are breast cancers, it seems completely counter to current understanding and dogma to represent breast cancers as rare diseases. Yet, from the earliest days of molecular analysis of breast cancer,^1^ through the molecular subclassification of breast cancers using transcriptomics^2^ to current multi-omics data,^3^ scientific advances are constantly challenging our view of breast cancer (and other diseases) as single entities. Clearly today, there exist multiple disease entities, defined at a molecular level but combined under the site of origin in the breast, some if not all of which may represent rare diseases.

### What is a rare cancer?

Definitions of “rare” cancers vary widely, even though many are based on a common statistical approach measuring annual incidence expressed per 100,000 individuals. Bodies including the World Trade Center health program (http://www.cdc.gov/wtc/pdfs/WTCHP_PP_RareCancers05052014.pdf/ http://epi.grants.cancer.gov/events/rare-cancers/) suggest incidence rates of 15/100,000 population/annum would be sufficient to define cancers as rare, whilst Rare Cancers Europe (http://www.rarecancerseurope.org/About-Rare-Cancers) and the European Society for Medical Oncology are more stringent, setting the rate at 6/100,000/annum. Most stringent are definitions set by the IRCI (http://ecancer.org/journal/editorial/20-international-rare-cancers-initiative-irci.php), which suggest rare cancers are confined to those with incidences of 2–3/100,000. Other definitions of “rare cancers” are less well characterized including “difficult to treat” cancers as “rare”.

### Could breast cancers be rare?

Breast cancer incidence in the US/Canada is around 155/100,000 women per annum, therefore if breast cancers were evenly subdivided in as few as 11 subgroups (using the broadest definition), these subgroups would individually represent “rare cancers”. Should the distribution of subtypes be uneven, as we would expect, the reality of rare breast cancers would become apparent with far fewer subdivisions. In fact, we can probably already identify rare breast cancer subtypes.

### Breast cancer subtypes–nothing new under the sun

Since the work of Perou and Sorlie et al. over 15 years ago,^2^ molecular subtyping of cancer has been both a driver of novel research approaches and an intense debating point in the clinical management of cancer. The identification of multiple subtypes presents a challenge both to researchers seeking to investigate breast cancer, and to clinicians seeking to treat this disease. Phrases like “one size does not fit all” or “divide and conquer” recognize this challenge intellectually but the question remains: Have we truly integrated the current knowledge base around multiple cancer subtypes and recognized the fundamental shift in both preclinical and clinical research strategy required for future progress in clinical management of breast cancer required by current evidence? We should note in passing that the work by Perou, Sorlie et al., however transformative, was far from the first to demonstrate the existence of “subtypes” in breast cancer. From the work of Beatson in 1890, through the development of targeted therapies such as anti-ER and anti-HER2 therapies from the 1960s–1990s, “subtyping” by oestrogen receptor (ER), PgR, and human epidermal growth factor receptor homologue 2 (HER2) measurement and other pathological features (e.g., classification of subtypes such as lobular carcinomas, etc) was central to both clinical management and preclinical understanding of breast cancer. This point is absolutely critical since it places Sorlie’s work correctly as a pivotal milestone in the continuous development of the understanding of the complex biology of breast cancer, rather than the final delineation of breast cancer subtypes. Recognizing this fundamental truth allows us to develop a broader molecular and functional mapping of the disease entities included under the umbrella of breast cancer and move towards a stratified and ultimately personalized medicine approach with the potential to rapidly impact patient management and survival.

### How many breast cancer sub-types?

Work on subtyping has progressed rapidly over the past few years, increasingly recognizing the need to integrate prior knowledge with novel approaches.

Illustrative of the molecular diversity within existing breast cancer subtypes is work investigating the molecular stratification of lobular breast cancers. Lobular cancers represent a subset of predominantly ER-positive breast cancers which are pathologically distinct from ductal carcinomas. They are also molecularly distinct from ductal ER-positive breast cancers, exhibiting in addition to CDH1 mutations/loss, markedly different mutational spectra to ER-positive ductal cancers. Pathologically and molecularly multiple subtypes of lobular breast carcinomas are identified.^4^ This may reflect differential clinical behavior of lobular breast carcinomas both in comparison to ductal carcinomas of the breast and within subsets of pathologically defined lobular carcinomas.

Likewise, broad molecular subgroups defined by Sorlie and others may be increasingly sub-divided with both clinical and molecular consequences. Amongst the 15–20% of newly diagnosed ER-negative breast cancers, approximately 50% are HER2-amplified and eligible for treatment with HER2 targeted agents. For the “triple negative” (ER, PgR, and HER2 negative) breast cancers, further sub-division into basal (CK5/6, EGFr expressing cancers) or 6 further molecular subgroups^5, 6^ also suggests a degree of complexity not fully explained by the first generation of molecular profiling experiments.

Finally, comprehensive molecular profiling approaches across multiple tumor types are providing further striking insights into molecular subtyping of cancers, suggesting classification of breast and other cancers across more than 30 molecular subclasses.^5, 7–9^

This evidence shows that breast cancer represents a far more complex panel of diseases than previously understood. Whilst for many approaches, the clinical utility of subtyping may require additional developmental work further sub-division of breast cancer appears inevitable. Whether we ultimately arrive at 10, 20 or as many as 50–100 sub divisions of disease, some, if not of most, subtypes will surely satisfy the definition of “rare cancers”.

### Does it matter?

The pivotal question is not how many subtypes of breast cancer exist, but rather what, if any, consequences the further subdivision of breast cancer might have on future clinical and preclinical research strategies? Put simply–are the strategies developed for evaluating novel treatment approaches when breast cancers was regarded as a single disease still viable and appropriate if breast cancer fragments into multiple cancer sub-types? Whether these cancers sub-types are rare or not may be secondary to the challenge of adapting research strategies to enable rapid progress towards a stratified and personalized medicine approach.

In the late 20th century, innovations in the adjuvant clinical management of breast cancer relied on ever larger phase III clinical trials seeking to identify increasingly marginal survival improvements for patients. Pivotal breast cancer trials over the past decade recruited many thousands of patients and were powered to detect modest (often 3–5%) improvements in absolute risk across these patients. Clearly such large trials are neither logical nor feasible in small cancer sub-groups as defined above. Indeed the entire approach to breast cancer trials might require re-evaluation.

To explore this question, let us accept that lobular cancers (ca 15% of all new breast cancers) represent at least 6 subtypes, that triple negative breast cancer represents a further 7 subtypes, and that 10–20 subtypes exist for ER positive breast cancers.^5, 7–9^ Let us further assume that these subtypes are evenly distributed within the cancer spectrum. Now, each of the 6 lobular carcinoma subtypes has an incidence of <4/100,000 per annum. For triple negative breast cancer (TNBC) subtypes incidence is <3/100,000. Only 13 subtypes of non-lobular luminal breast cancers are required for these to have incidences of <6/100,000 per annum. Each subtype would then represent between 4500–9000 new cases in the US in 2016 (based on statistics at http://www.breastcancer.org/), roughly equivalent to the number of new testicular cancers. Clearly in this context, existing trial designs and approaches would not be logical, unless we persist in ignoring the message of molecular diversity in breast cancer.

## Learning from rare cancers?

Rare cancers are recognized as challenging to treat, in part simply because generating robust evidence for changes in clinical management becomes increasingly challenging when faced with small numbers of cases. There are statistical, clinical, organizational, and financial challenges to performing clinical trials in this space which are outside the scope of this review. However, the innovative clinical trials approaches developed by experts in the field of rare cancers are likely to be of increasing value to those researching rare sub-types of “common” cancers. These novel approaches are excellently surveyed by Bogaerts et al.^10^ providing a starting point for addressing smaller sub-groups of cancers using robust clinical trial methodology. These approaches are already, in some instances, being applied in breast cancer^11, 12^ and other “common” but molecularly diverse tumor types (http://www.focus4trial.org/).

The framework for clinical research in this context is therefore increasingly applied to molecular sub-grouping within multiple common cancers. There exist clinical trial strategies, and a framework for those being extended, to addressing multiple sub-types simultaneously or select individual sub-groups for small, focused, studies. The challenge remaining is to operationalize innovations across the spectrum from basic research to clinical trial design to maximize progress in evaluation of novel therapeutics for patients.

## Stratified medicine for breast cancer–an evidence based strategy

**Fig. 1:**
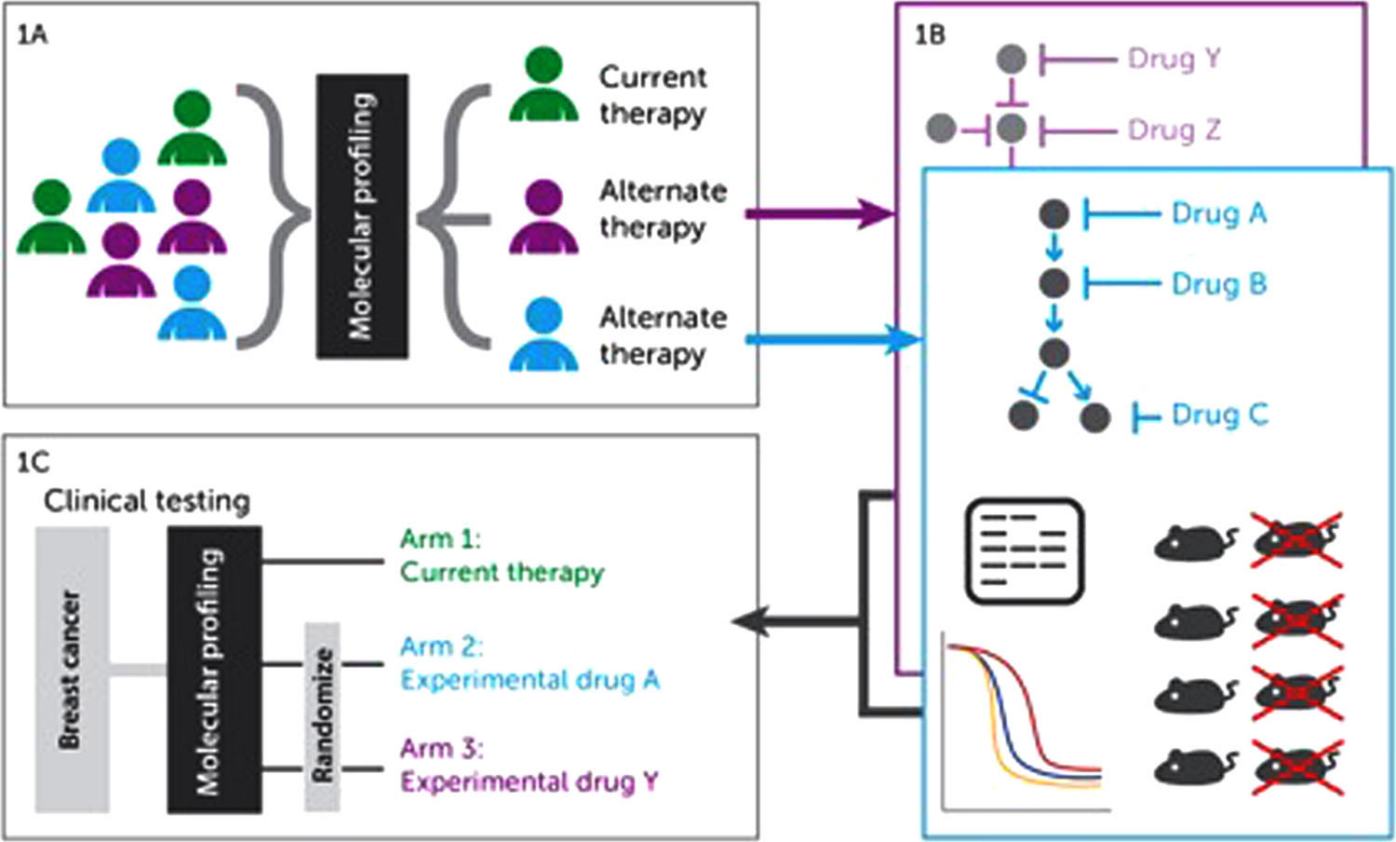
Schema for addressing multiple breast cancer disease entities through integrated diagnostic assay, targeted therapeutic, and clinical trial strategies. Panel **a**: Molecular stratification to identify treatment refractory tumors; ideally performed within unified patient cohorts or clinical trial biobanks. *Green* represents patients at low residual risk following standard of care (eligible for future monitoring or treatment reduction trials). *Purple/blue* patients are those with treatment refractory tumors with candidate molecular drivers. Panel **b**: Overview of preclinical validation for pathway drivers. Given extensive targeted drug portfolios repurposing of existing agents may act as a further accelerator to clinical validation. Panel **c**: Draft clinical trial schema–matching molecular strata (*purple/blue*) to repurposed drugs

How might such novel clinical trial approaches be applied in the context of breast cancer? There are multiple potential approaches, which could be applied across adjuvant, neo-adjuvant or metastatic settings. Fig. 1 outlines a high level example strategy, which is neither comprehensive nor exclusive of other approaches. It is best viewed as a “straw dog”–an idea set up to be modified and to frame a debate. Many readers will recognize the key steps intuitively and be able to adapt and apply the broad concepts outlined. The objective is integrate key steps required to validate a molecularly stratified treatment approach for different breast cancer entities. Clearly, however, the delivery of a successful clinical trial in this setting requires a molecular approach to stratification that is both informative, in the context of existing treatment, and actionable by linking to novel treatment opportunities.

### Increasing predictive value of molecular profiling

Many existing approaches to molecular profiling have focused primarily on prognostic, rather than predictive outcomes. They allow the segregation of patients into high or low risk groupings in the context of existing therapies, but provide minimal information to inform the appropriate treatment intervention which might reduce relapse in those for patients at high risk. In addition, most tests treat all high risk patients as representing a single group. However, the complexity of breast cancer suggests patients at high risk of progression include those from multiple breast cancer subtypes each of which may exhibit specific but different molecular drivers which underpin treatment failure. Future molecular profiling approaches should recognize this challenge and seek to provide functional means of segregating patients at high risk, (Fig. 1a) who are most in need of alternate therapies, into groupings mapped against key molecular drivers/targets. This approach is a logical extension of the molecular stratification used to identify the HER2 oncogene as a potential treatment target. Novel approaches, such as those applied in TNBC by Lehmann et al.,^5^ serve to demonstrate the continued value of clinical cohorts to develop such approaches. The wealth of clinical trial tumor banks established in breast cancer provide the ideal setting for such approaches, mapping molecular events to specific and current treatment outcomes.

### Mapping casual to causal/actionable targets

As with HER2, identifying a casual relationship between outcome/treatment failure and a molecular driver or candidate provides clues, not validated targetable pathways. This is a critical step which cannot be bypassed. Therefore, for each identified subtype or “strata” robust preclinical evidence mapping the optimal therapeutic agent with maximum potential to improve outcome within the selected group is required (Fig. 1b). Only if such evidence is developed can clinical interventions be planned which map to the treatment and molecular context of the specific disease with a high probability of success.

### Integrating molecular profiling and targeting in future trials

The critical challenge will be to link molecular profiling to actionable variants and to apply these within the context of multi-arm, molecularly stratified clinical trials. These trials will need to integrate and extend concepts already applied within existing trials (e.g., FOCUS4 and I-SPY2) extending them to encompass novel molecular approaches to stratification based on functionally validated targets. Key components could include (Fig. 1c); identification of patients at low risk of relapse to be monitored in an observational arm excluded from randomization. Stratification using similar, preferably identical, assay criteria to those used to identify original molecular strata (Fig. 1a). Randomization within treatment arms to adjust for differential outcome between molecular subgroups. Bayesian statistics to adjust for smaller sample sizes within arms and robust biomarker development strategies to further refine patient selection.

## Summary and conclusion

Molecular profiling of breast cancers strongly supports the further fragmentation of this disease into multiple molecular sub-types or strata. Increasingly, molecular targeting of key mutational drivers provides a rapidly expanding armamentarium of clinically testable agents for molecular targeting (e.g., ref. 13). The challenge remains for researchers, across the scientific spectrum, to implement appropriate clinical and pre-clinical approaches to rapidly translate the wealth of molecular information. Amongst other challenges in this area these are those which are regarded as the most critical barriers to progress:

1. Development of robust diagnostic approaches to the molecular stratification of cancers: Existing approaches to molecular stratification of breast cancer have focused almost entirely on prognostic, rather than predictive, tests. Future stratified medicine trials will require validated molecular profiles, which can be implemented in routine diagnostic laboratories, focusing on actionable molecular events/pathways in the context of current clinical therapeutics, to select those patients most likely to require and respond to novel therapeutics. This area is perhaps the most under-resourced in current stratified medicine approaches.
2. Appropriate validation of targeted therapeutics within molecular strata: Given the large number of targeted therapeutic agents currently under evaluation, with multiple agents per target/pathway, selection of the best agents is essential. Use of agents at appropriate doses, keeping the targeting “clean”, and in the appropriate population requires careful selection of strategies for pre-clinical evaluation.
3. Optimal clinical trial design and patient screening: Molecular stratification should be regarded as an essential component of future clinical trial design, unless strong arguments to the contrary can be made. Such stratification should achieve two separate goals. Firstly, to screen out patients at such low risk of disease progression that current conventional treatment results in effective disease control. Secondly, to allow responses to be evaluated prospectively within molecular strata in which appropriate matching between molecular drivers and targeted therapeutics is optimized.

Knowledge regarding the molecular complexity of breast and other tumors has transformed our understanding of cancer. Research approaches need to adapt to address this new challenge. Rapid expansion in both the availability of novel targeted therapeutics and the identification of molecular alterations in cancers represent a significant challenge to the research community to accelerate approaches which address molecularly distinct sub-types of cancer. This review suggests some key challenges which must be addressed. It is intended to spur debate and discussion rather than to offer a final solution.
